# Surface deformation analysis of collapsed lungs using model-based shape matching

**DOI:** 10.1007/s11548-019-02013-0

**Published:** 2019-06-27

**Authors:** Megumi Nakao, Junko Tokuno, Toyofumi Chen-Yoshikawa, Hiroshi Date, Tetsuya Matsuda

**Affiliations:** 1 Graduate School of Informatics, Kyoto University, Yoshida-Honmachi, Sakyo-ku, Kyoto, Japan; 2 Department of Thoracic Surgery, Kyoto University Hospital, 54 Kawaharacho, Shogoin, Sakyo-ku, Kyoto, Japan

**Keywords:** Pneumothorax deformation analysis, Model-based shape matching, Lung, Thoracoscopic surgery

## Abstract

**Purpose:**

To facilitate intraoperative localization of lung nodules, this study used model-based shape matching techniques to analyze the inter-subject three-dimensional surface deformation induced by pneumothorax. Methods: Contrast- enhanced computed tomography (CT) images of the left lungs of 11 live beagle dogs were acquired at two bronchial pressures (14 and 2 cm$\,\hbox {H}_2\hbox {O}$). To address shape matching problems for largely deformed lung images with pixel intensity shift, a complete Laplacian-based shape matching solution that optimizes the differential displacement field was introduced.

**Results:**

Experiments were performed to confirm the methods’ registration accuracy using CT images of lungs. Shape similarity and target displacement errors in the registered models were improved compared with those from existing shape matching methods. Spatial displacement of the whole lung’s surface was visualized with an average error of within 5 mm.

**Conclusion:**

The proposed methods address problems with the matching of surfaces with large curvatures and deformations and achieved smaller registration errors than existing shape matching methods, even at the tip and ridge regions. The findings and inter-subject statistical representation are directly available for further research on pneumothorax deformation modeling.

**Electronic supplementary material:**

The online version of this article (10.1007/s11548-019-02013-0) contains supplementary material, which is available to authorized users.

## Introduction

Recent advances in medical imaging technology have enabled visualization of early stage cancer, metastatic lung tumors, and benign nodules. Video-assisted thoracoscopic surgery [1, 2] is a widely performed minimally invasive surgical procedure. Although lung nodules are examined on preoperative computed tomography (CT) images during preoperative planning, the position of a nodule may change because of the state of pneumothorax during surgery, which makes optimization of resection procedures difficult. Although various attempts have been made to use physical or chemical markers [3, 4] to identify the varying positions of multiple nodules, the associated clinical burdens on both surgeons and patients are increased because of the additional CT imaging and preoperative marking procedures required. If the intraoperative positions of lung nodules could be accurately estimated, precise nodule resection and preservation of pulmonary function could be facilitated by the strict management of resection margins.

Deformable image registration techniques [5–7] for the analysis of organ and soft tissue deformations have been explored previously. Intraoperative deformation due to changes in internal pressure, patient posture, and tool manipulation is a well-known practical registration problem, which must be addressed in the development of intraoperative guidance systems [8–16]. Specifically, in the field of image-based lung modeling, respiratory motion has been the main focus of investigation [17–21]. However, there have been few studies on modeling of the pneumothorax deformation that occurs between the preoperative and intraoperative lung states. Shape matching of the pneumothorax deformation of the lung should address the technical issues of the large deformations and CT intensity shifts that occur. Lungs are very soft organs, and their deformation can induce considerable volume change. The mechanism of pneumothorax deformation is complex and not mathematically understood, except through simulation studies of animal lungs [17, 22]. CT intensity shifts occur in the atelectasis state, where the air content of the lungs is reduced, resulting in changes to CT values and lowering of image contrast, and reduced performance for image-based registration.

To localize lung nodules during surgery, Nakamoto et al. [23] proposed intraoperative registration methods that matched the surface of a preoperative CT model with several surface points optically measured on the deflated lungs. Intraoperative segmentation of thoracoscopic camera images was investigated in [24], and Alvarez et al. recently reported deformable registration results for lung deformations resulting from postural differences on cone beam CT (CBCT) [25]. Uneri et al. also proposed a registration framework to analyze the displacement of internal lung structures on CBCT data from animal lungs [26]. To address CT intensity shifts, an integrated framework of model-based and image-based registration was used; however, registration of the bronchial branch points was the main focus, and registration accuracy at the lung surface was not evaluated.

The goals and clinical needs of intraoperative guidance using video-assisted thoracic surgery (VATS) are visualization of the subsurface positions of nodules, bronchial structures, and vascular structures in the collapsed lungs. However, it is difficult to image internal structures with VATS, because CBCT imaging is not available in most clinical facilities, and it may increase the burden on patients and surgeons. Recent studies [12, 27] proposed using the 2D appearance or silhouettes of organs as visual cues to register preoperative CT models with intraoperative camera images. We also consider that the intraoperative appearance of collapsed lung surfaces could be an essential visual cue [28, 29]. Clinical applications of surface deformation models are widespread and include initial model alignment and 2D–3D surface matching, e.g., matching of 2D intraoperative camera images (2D surface or silhouettes) with volumetric deformation models (3D surface with subsurface structures) of the collapsed lungs. Integrating surface and subsurface deformation models into a thoracoscopic camera image recognition system could lead to novel vision-based guidance for VATS, which could provide subsurface nodule visualization without additional CBCT measurement. However, to the best of our knowledge, no study has shown such registration results, or visualized inter-subject variation in spatial deformation of whole lung structures. Specifically, registration error tends to increase in areas with substantial curvature, such as the tips of the lobes or the boundary region between the upper and lower lobes. Therefore, data acquisition and detailed surface analysis is worth investigating in the search for a statistical formulation of lung deformation.

In this study, we used model-based shape matching to analyze three-dimensional surface displacement in collapsed lung. First, CT datasets were acquired at two bronchial pressures (assigned as the inflated and deflated states) in 11 live beagle dogs. Although spatial distribution of the displacement of internal structures can be obtained by the matching of anatomical feature points such as bronchial branches [23, 26], landmark-based matching cannot be applied to curved surfaces, because of their lack of anatomical features. As the acquired CT data may contain large deformations with CT intensity shifts, global image registration techniques [30] tend to lead to large registration errors, particularly at tip regions with high curvature.

The practical applications of model-based registration are increasing, as registered mesh models are directly available for statistical modeling and variational analysis [31–33]. In addition, a recent study [32] reported that registration accuracy for anatomical structures with large shape variations was better than that obtained using large deformation diffeomorphic metric mapping [7]. Here, we employ a model-based shape matching approach utilizing improved Laplacian-based shape matching (LSM) techniques for pneumothorax deformation. We performed experiments to confirm the performance of the proposed shape matching method using CT data and to summarize the spatial distribution of the three-dimensional displacement of the lung surface during pneumothorax deformation.

The contributions of this paper are: (1) a complete model-based surface registration solution that optimizes a differential displacement field to map large pneumothorax deformations and (2) analysis of the spatial displacement of whole inflated/deflated lungs, including statistical deformation model representations. This paper does not focus on discussing the technical aspects of the registration methods, but instead concentrates on reporting the findings of pneumothorax deformation analysis with application of improved model-based shape matching.

## Methods

### Measurements and surface reconstruction

To analyze the deformation of collapsed lung, contrast-enhanced CT images of the left lungs of 11 live beagle dogs were acquired at two bronchial pressures (14 and 2 cm $\hbox {H}_2\hbox {O}$) at the Institute of Laboratory Animals, Kyoto University. This study was performed in accordance with the regulations of the Animal Research Ethics Committee of Kyoto University. All CT images were acquired on a 16-row multidetector CT scanner (Alexion 16, Toshiba Medical Systems, Tochigi, Japan). During the procedure, the dogs were maintained under anesthesia with ketamine, xylazine, and rocuronium and underwent tracheal intubation and mechanical ventilation by a ventilator (Savina 300, Drager AG & Co. KGaA, Lübeck, Germany). A single trocar hole was first made on the chest wall, to let air flow into the pleural cavity. Using the ventilator, the bronchial pressure was set to 14 cm$\hbox {H}_2\hbox {O}$ to obtain images of the fully expanded lungs (inflated state), and to 2 cm$\hbox {H}_2\hbox {O}$ for imaging of the collapsed lung state (deflated state). All dogs were placed in a right lateral (decubitus) position on the bed of the CT scanner, and the two CT image sets of the inflated and deflated states were acquired in that order for each dog. For the contrast-enhanced CT, 10 mL of iopamidol contrast agent was injected through a lower extremity peripheral vein. Scanning was performed 5 s after the injection of iopamidol.

**Fig. 1:**
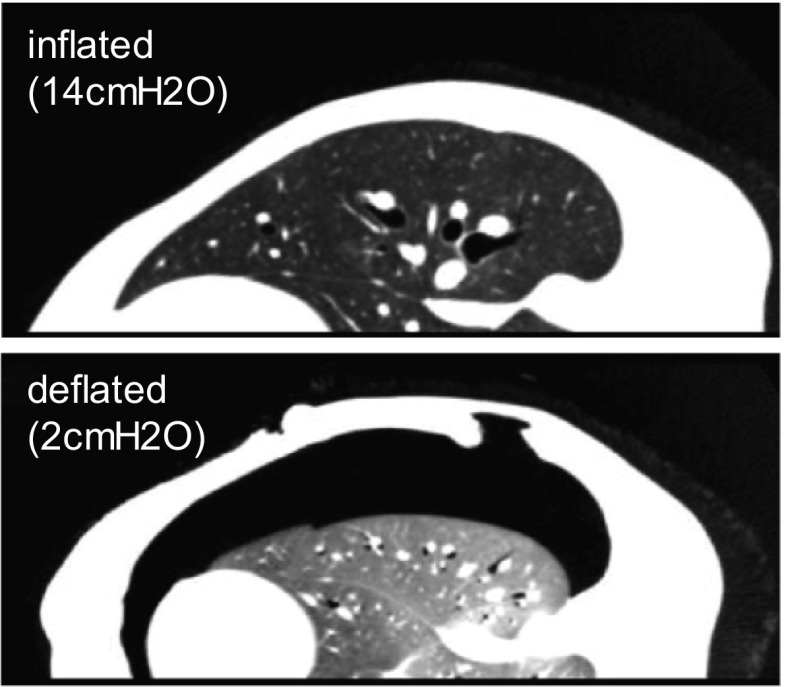
CT images of inflated/deflated states with intensity shift measured from the left lung of a live beagle dog

Figure 1 shows CT slices from the inflated and deflated states after registering the two volumes using the spine as a fixed reference. The CT intensity values change because of differences in the air content of the lung, with the CT intensity values of the parenchyma increasing (the parenchyma region becomes brighter) in the deflated state. This intensity shift can be clearly observed in the lower image of Fig. 1 when it is compared with the upper image. For details, refer to [26]. The registration accuracy of image-based registration can be affected by these imaging characteristics of pneumothorax deformation, as most of the methods assume consistent pixel features. The proposed model-based registration approach achieves stable registration for pneumothorax deformation analysis on the basis of surface geometry.

Anatomical segmentation of the upper and lower lobes was automatically performed using the Synapse VINCENT image analysis system (Fujifilm Co., Ltd.), and the surfaces of the lobes were generated as triangulated mesh representations using Poisson surface reconstruction [34]. The meshes of the two lobes were created independently, and each triangulated mesh was stored in the standard STL or PLY file format.

**Fig. 2:**
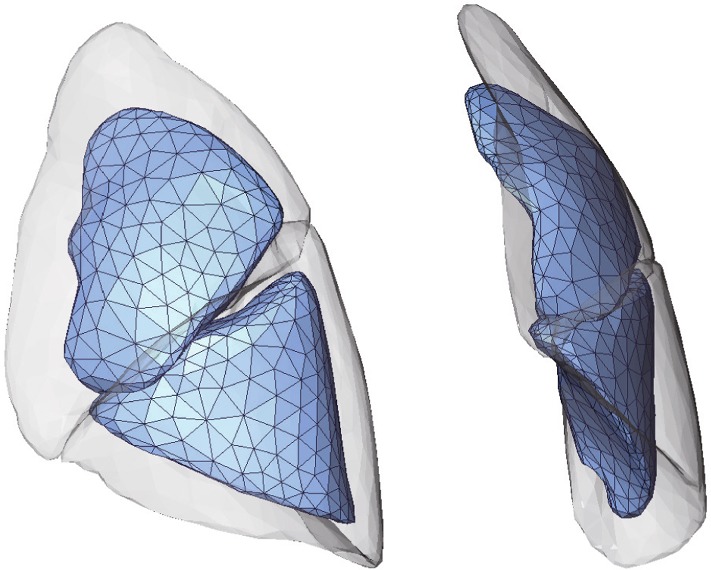
Example of reconstructed lung surfaces in inflated and deflated states. The translucent image depicts the inflated state, and the opaque image with the mesh topology is the deflated state

### Shape matching between inflated and deflated lungs

**Fig. 3:**
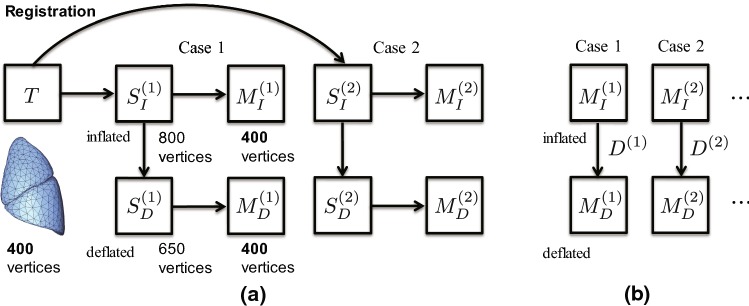
Outline of the inter-subject shape matching framework. **a** Corresponding models $(M_{I}^{(k)}$, $M_{D}^{(k)})$ with the same vertex and the same mesh topology are computed by registering the template *T* to the individual surfaces $(S_{I}^{(k)}$, $S_{D}^{(k)}$). **b** The spatial deformation $D^{(k)}$ between the inflated state and deflated state is obtained from the registration between the two models

To calculate the lung surface displacement, shape matching was performed on the triangulated meshes reconstructed from the CT images. Figure 2 shows an example of reconstructed lung surfaces in the inflated and deflated states, and Fig. 3 outlines the inter-subject shape matching framework developed using the statistical motion modeling expression [33]. A template surface *T* was used as the source, and the individual surfaces $S_{I}^{(k)} (k=1, 2,\ldots , n)$ in the inflated and $S_{D}^{(k)} (k=1, 2,\ldots , n)$ deflated states were used as the targets. In this study, *n* equals 11, because we prepared an image dataset with 11 subjects in the inflated and deflated states. Here, we assume that the pair of surfaces $(S_{I}^{(k)}$ and $S_{D}^{(k)})$ differs in the number of vertices and the structure of the mesh (i.e., mesh topology), as they were independently generated from different CT images. As shown in Fig. 3a, the corresponding models $M_{I}^{(k)}$, $M_{D}^{(k)}$ (with the same vertex and the same mesh topology) that precisely approximated the surfaces $S_{I}^{(k)}$ and $S_{D}^{(k)}$, respectively, are computed by shape matching. Because the two registered models achieve point-to-point correspondence, spatial deformation $D^{(k)} = M_{D}^{(k)} - M_{I}^{(k)}$ can be represented by calculating the displacement vector of the corresponding vertex, as shown in Fig. 3b. To capture rotational components or the sliding motion of the upper and lower lobes, this registration process is applied to each lobe independently. Unlike per-subject registration, our approach enables the construction of a statistical deformation model, making deformation analysis among subjects possible.

For the template generation, one case was randomly selected and set as the initial surface for *T*, and the triangular surface was resampled to 400 vertices and 796 triangles for each lobe. Next, the corresponding models ${M}_{I}^{(k)}$ were obtained by registering *T* to the individual surfaces $S_{I}^{(k)}$ in the inflated state. As the mesh models ${M}_{I}^{(k)}$ have point-to-point correspondence, the average shape $\overline{M}$ can be obtained by calculating the average of each coordinate. We used $\overline{M}$ as the final template. By keeping the template close to the data to be matched in advance, we aimed to reduce the influence of the template shape’s data selection method on the matching, while preventing increased matching error.

Accurate shape matching is required to compute a stable three-dimensional displacement field from the substantially deformed surfaces. As pneumothorax deformation includes considerable volume changes and rotations, the registration error of image-based global registration methods increases in areas with large curvature, such as the tips of the lobes or boundary region between the upper and lower lobes. To achieve both globally stable and locally strict registration, Laplacian-based shape registration is used after executing affine transformation. A discrete Laplacian was first introduced for interactive editing of a geometric model [35] and was recently applied to non-rigid shape modeling. In [32], Laplacian-based registration showed better registration performance than large deformation diffeomorphic metric mapping [7] for curved surfaces with shape variations. Lung surfaces also move considerably during pneumothorax deformation, and the registration accuracy is not understood. Therefore, in this research, we extended the Laplacian-based registration technique to shape matching of inflated/deflated lungs with large-scale deformations and investigated its registration performance.

### Laplacian-based surface registration using a differential displacement field

The overall process of the shape matching framework developed is described as follows.

1. A discrete Laplacian $L({\varvec{v}}_{i})$ and a normal vector ${\varvec{n}}_{i}$ are calculated for all vertices ${\varvec{v}}_{i}$ of the template *T* and the target surface *S*.
2. Localized shape similarity $Q_{i}$ between the template and the target surface is calculated for all vertices of the template mesh.
3. The positional constraint ${\varvec{p}}_{i}$ for shape update is determined based on Eq. (4).
4. The new positions for the set of vertices ${\varvec{v}}'_i$ are calculated based on Eq. (2). The shape is updated as ${\varvec{v}}_{i} \leftarrow {\varvec{v}}'_{i}$. Then, back to STEP 1.

The discrete Laplacian obtained in STEP 1 is a shape descriptor defined as Eq. (1) that approximates the mean curvature normal of the triangular mesh.

1 $$\begin{aligned} L({\varvec{v}}_{i}) = \sum _{j \in N({\varvec{v}}_{i})} \omega _{ij} ({\varvec{v}}_{i}- {\varvec{v}}_{j}) \end{aligned}$$

Here, $\omega _{ij}$ is a weight, and $N({\varvec{v}_i})$ is the number of adjacent vertices of one ring connected by the vertex ${\varvec{v}_i}$ and the edge.

2 $$\begin{aligned} \hat{V} = \mathop {\mathrm{arg~min}}\limits _{V} \sum _{i=1}^{n} \Vert L( {\varvec{v}}'_{i})\ - L({\varvec{v}}_{i}) \Vert ^{2} + \delta \sum _{i =1}^{n} \Vert {\varvec{p}}_{i} - {\varvec{v}}_{i} \Vert ^{2} \nonumber \\ \end{aligned}$$

where *V* is the set of vertices ${\varvec{v}_i}$ at their initial positions, and $V'$ is the set of vertices ${\varvec{v}'_i}$ to be solved. ${\varvec{p}_i}$ is a positional constraint set to ${\varvec{v}_i}$, and $\delta$ is a weight parameter configured according to the problem. $L(\cdot )$ is the Laplace–Beltrami operator, and $L({\varvec{v}_i})$ is the discrete Laplacian at the vertex ${\varvec{v}_i}$. The first term is a penalty to shape changes to the mesh, and the second term increases if the constrained vertex is distant from the target position ${\varvec{p}_i}$. By computing ${\varvec{v}'_i}$, which minimizes the objective function, the template model is updated while preserving the shape as much as possible. Because Eq. (2) is a quadratic minimization problem at vertex positions ${\varvec{v}_i}$, it is possible to calculate it with a low computation cost.

Here, we describe the method of determining the positional constraints ${\varvec{p}_{i}}$ in STEP 2 and STEP 3. It is difficult to find appropriate positional constraints in the initial state when the deformation between surfaces is large and anatomical landmarks and point-to-point correspondences are not given. Therefore, we explored better constraint options by performing a progressive search and additionally introduced a new definition of positional constraints that enabled us to obtain smooth displacement while performing precise surface matching. To obtain the three-dimensional position on the target model corresponding with the vertex of the template model, the shape similarity value *Q* was calculated for all combinations of the vertex $v_{s}$ of the template model and the vertex $v_{t}$ of the target model. *Q* is defined by (3)

3 $$\begin{aligned} Q(\varvec{v}_s, \varvec{v}_t) = | \varvec{v}_s - \varvec{v}_t | + \gamma ( 1 - \varvec{n}_s \cdot \varvec{n}_t ) \end{aligned}$$

where $\gamma$ is a weight, and ${\varvec{n}_s}$, ${\varvec{n}_t}$ are vertex normals in ${\varvec{v}_s}$, ${\varvec{v}_t}$, respectively. *Q* locally evaluates shape similarity per vertex, and if *Q* is small enough, the pair of tangent planes expressed by ${\varvec{v}_s}$ and ${\varvec{v}_t}$ is corresponding surfaces.

**Fig. 4:**
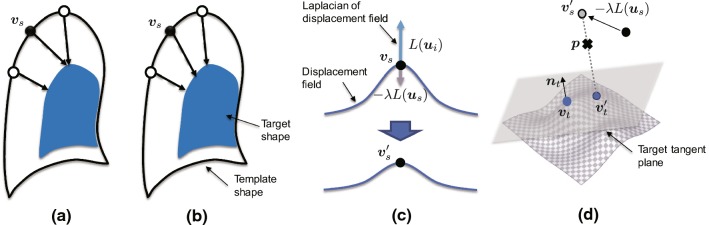
Positional constraints for Laplacian-based surface registration to ensure a smooth differential displacement field. When the corresponding pair of vertices $({\varvec{v}}_{s}, {\varvec{v}}_{t})$ is given, the positional constraint ${\varvec{p}}$ is determined as an internal division point of the smoothed position ${\varvec{v}}'_{s}$ and its projected position ${\varvec{v}}'_{t}$ on the target tangent plane

The positional constraints ${\varvec{p}}_{i}$ are configured on the basis of the local similarity *Q* for all vertices of the template model. For a group of vertices whose *Q* is small enough, or close to zero, the positions of vertices ${\varvec{v}}_{s}$ are maintained, that is, ${\varvec{p}}_{i} = {\varvec{v}}_{s}$ is given, because they adequately represent the local surface of the target shape. Alternatively, for a group of vertices with a large *Q* value, a positional constraint is set to correct the local shape of the template. In this case, if the corresponding point is determined independently for each vertex of the template model, the progressive shape update may yield a non-smooth displacement field. Figure 4a briefly illustrates the issue of local matching error caused by independently configured positional constraints. To address this problem, we focused on the differential displacement field $\nabla {\varvec{u}}$ and achieved smooth deformation while performing precise surface matching. Figure 4b provides an example of the modified positional constraints obtained after shape matching.

The differential displacement field is computed by the set of partial differentials of displacements ${\varvec{u}}_{s}$ on the vertex ${\varvec{v}}_{s}$. To smooth the displacement field, the changes in the gradients of the displacement field ${\varvec{u}}_{s}$ should be kept small, as shown in Fig. 4c. This means that smooth deformation matching between two shapes can be performed by minimizing the Laplacian of the displacements $L({{\varvec{u}}_{s})}$. This concept is based on Laplacian-based mesh optimization [36], which achieves feature-preserving smoothing of triangular meshes. The different aspects are that this strategy (1) applies its basic technique to the displacement field, not to the surface, and (2) undergoes progressive updates throughout the shape matching process, which aims to solve the trade-off problems of the nearest neighbor search between point-to-point correspondences and maintenance of a smooth displacement field.

On the basis of this scheme, when the pair of corresponding vertices $({\varvec{v}}_s, {\varvec{v}}_t)$ is determined by the local similarity index *Q*, the positional constraint ${\varvec{p}}$ is determined as an internal division point of the smoothed position ${\varvec{v}}'_{s}$ and its projected position ${\varvec{v}}'_{t}$ on the target tangent plane. Figure 4d illustrates the setup of positional constrains ${\varvec{p}}_{i}$ from the pair of vertices $({\varvec{v}}_s, {\varvec{v}}_t)$. This step-by-step update avoids local mismatch at the early stage assuming a considerable distance between the two surfaces. Consequently, the positional constraints $\varvec{p}_{i}$ are defined by Eq. (4).

4 $$\begin{aligned}&\varvec{p}_{i} = \left\{ \begin{array}{ll} \varvec{v}'_{s} + \dfrac{l_{s}}{m} ({\varvec{v}}'_{t} - \varvec{v}'_{s}) &{} ( Q > Q_\mathrm{high})\\ \varvec{v}'_{s} &{} ( Q < Q_\mathrm{low} ) \end{array} \right. \end{aligned}$$

5 $$\begin{aligned}&\varvec{v}'_{s} = \varvec{v}_{s} - \lambda L({\varvec{u}}_{i}) \end{aligned}$$

6 $$\begin{aligned}&L({\varvec{u}}_{i}) = \sum _{j \in N({\varvec{v}}_{i})} \omega _{ij} ({\varvec{u}}_{i}- {\varvec{u}}_{j}) \end{aligned}$$

Here, $l_{s}$ is the average length of all edges connected to the vertex ${\varvec{v}}_{i}$, and *m* is a step constant for the progressive search. The two threshold values $Q_\mathrm{high}$ and $Q_\mathrm{low}$ affect the stability of the shape update and the convergence speed. For example, if a high value is used for $Q_\mathrm{high}$, a smaller number of vertices are constrained at the target surface, which results in slower convergence. When a low value is used for $Q_\mathrm{low}$, the template shape becomes easily deformable, possibly destabilizing the shape updating, as a smaller number of vertices are fixed. In our case, after investigating various parameter sets, the average value of the top 10% was used as $Q_\mathrm{high}$, and the average value of the bottom 2% was used as $Q_\mathrm{low}$.

In STEP 4, the template surface is updated by applying the positional constraint ${\varvec{p}}$ to the quadratic minimization equation (2). The shape optimization defined by STEP 1 to STEP 4 is iteratively processed. When the maximum value of the inter-surface distance between the template and the target model is not improved when the surface update is repeated ten times, or when the number of updates reaches 3000 times, the iterative process is terminated. In this framework, even when the number of vertices differs between the template and target models, or even when the vertex of the target model does not exist near to the corresponding local region, the optimized matching of corresponding local surfaces can be computed.

## Experiments and results

In the experiments, the performance of the proposed Laplacian-based shape matching framework was first evaluated. The efficacy of the differential displacement field was confirmed by comparing it with existing shape registration methods. Then, the pneumothorax deformation of lungs was investigated in terms of the linearity and morphological variation of deformation using the registered models. The proposed shape matching framework was implemented using Visual C/C++ and OpenGL. A computer with a graphics processing unit (CPU: Intel Core i7 3.7 GHz, Memory: 64 GB, GPU: NVIDIA GeForce GTX 1080) was used throughout the experiments. For the weights in the developed framework, we used 10.0 for $\delta$, and 1.0 for $\omega$, $\delta$ and $\gamma$ after examination of several parameters sets.

### Performance evaluation

Shape matching was performed on 11 in vivo lung models (Cases 1–11) to confirm registration accuracy. The procedure for creating the template *T* and lung mesh models from the in vivo CT images is described in “Measurements and surface reconstruction section.” Table 1 summarizes the volumes of the upper/lower lobes at bronchial pressures of 14 and 2 cm$\hbox {H}_2\hbox {O}$ for each case, with *V* being the volume. For the superscript/subscript characters, *u*: upper lobe, *l*: lower lobe, *i*: inflated state, and *d*: deflated state. *r* is the ratio of volume change: $V^{d}/V^{i}$. We note that the volume of Case 11 at a bronchial pressure of 2 cm$\hbox {H}_2\hbox {O}$ was unexpectedly increased, which is probably a result of the bronchial pressure not being successfully controlled. However, as shape matching can be applied to such an inflated shape, the performance of the shape matching was evaluated for all the datasets.

**Table 1:** Volumes of upper/lower lobes on CT images of the left lungs of live beagle dogs. *V* is the volume

Case	$V_{u}^{i} [cc]$	$V_{u}^{d} [cc]$	$r_{u}[\%]$	$V_{l}^{i} [cc]$	$V_{l}^{d} [cc]$	$r_{l}[\%]$
1	173.5	82.7	47.7	242.9	96.9	39.9
2	167.1	69.9	41.8	508.3	116.4	22.9
3	231.7	111.4	48.1	357.6	145.3	40.6
4	184.7	71.4	38.7	258.2	100.4	38.9
5	246.1	78.9	32.5	353.3	92.8	26.3
6	188.4	65.8	34.9	373.2	104.1	27.9
7	131.9	55.8	42.3	257.9	110.7	42.9
8	164.3	102.3	62.2	342.1	196.7	57.5
9	198.8	84.9	42.7	375.4	111.8	29.8
10	232.5	113.5	48.8	355.7	186.4	52.4
11	163.7	160.2	97.9	212.1	236.6	111.5
Mean ± SD	189.3 ± 35.1	90.6 ± 29.7	48.9 ± 18.1	330.6 ± 83.4	136.2 ± 48.7	44.6 ± 24.7

Superscript/subscript characters: *u*: upper lobe, *l*: lower lobe, *i*: inflated state, and *d*: deflated state. *r* is the ratio of volume change: $V^{d}/V^{i}$

**Fig. 5:**
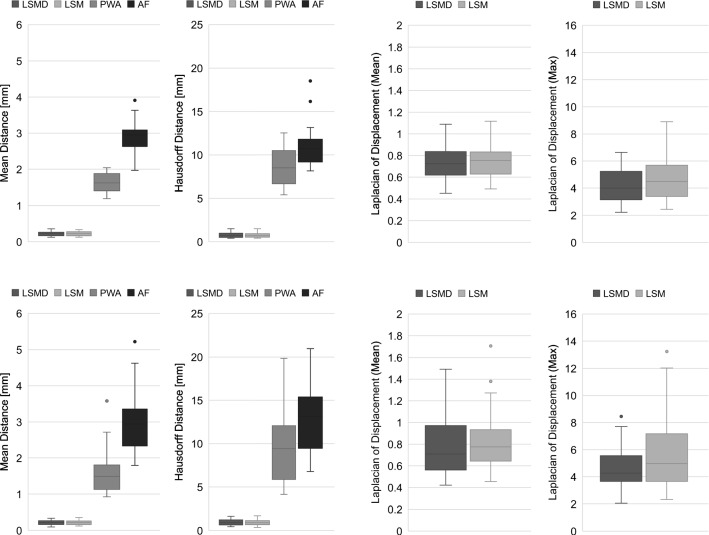
Quantitative comparison results: mean distance, Hausdorff distance, and Laplacian of the displacement (mean and maximum) of shape matching algorithms for inflated (upper row) and deflated (lower row) lungs

#### Quantitative comparisons of shape matching

In this study, the mean distance [32], Hausdorff distance [37], and Laplacian of the displacement were used as the shape similarity criteria. The Hausdorff distance measures the longest distance among minimum point distances between two surfaces, whereas the mean distance is the average of the minimum point distances. Unlike segmentation or recognition problems, shape matching requires point-to-point correspondence between two shapes. For example, as the Dice coefficient only measures volume overlap, it is not sufficient to evaluate per-vertex correspondence, nor to measure the quality of local matching. The Laplacian of the displacement is the magnitude of the second derivatives of the displacement field and evaluates the smoothness of the deformation. The registration accuracy was compared among four shape matching approaches: (1) LSMD: the proposed Laplacian-based shape matching with optimization of the differential field defined in this paper, (2) LSM: Laplacian-based shape matching with a similar progressive deformation approach used in [32] and [38], (3) PWA: piecewise affine transformation [39], and (4) AF: affine transformation. For all algorithms, affine transformation was performed in advance, to globally match the posture and volume of the entire shape.

Figure 5 shows box plots of the mean distance, Hausdorff distance, and mean and maximum Laplacian of the displacement computed from the 11 subjects’ registration results. Figure 5a, b shows the results for upper lobes and lower lobes, respectively. The box plots include the minimum, first quartile (*Q*1), median (*Q*2), third quartile (*Q*3), and maximum. The minimum and maximum scores are represented after outliers were rejected. Values larger than $(Q3-Q1) \times 1.5 + Q3$ or smaller than $Q1 - (Q3 - Q1) \times 1.5$ were regarded as outliers. The average, minimum, and maximum values of all datasets are summarized for LSMD, LSM, PWA, and AF in Table 2.

**Table 2:** The average (minimum and maximum) values of all shape matching results for mean distance (MD), Hausdorff distance (HD), Laplacian of displacements (LD), and target displacement error (TDE)

Metic	Methods			
	LSMD	LSM	PWA	AF
MD	0.22 (0.10–0.36)	0.22 (0.12–0.36)	1.6 (0.92–3.58)	3.0 (1.79–5.21)
HD [mm]	0.85 (0.39–1.63)	0.82 (0.36–1.70)	9.16 (4.14–19.8)	12.0 (6.78–21.0)
LD (mean) [mm]	0.76 (0.42–1.49)	0.79 (0.45–1.70)		
LD (max) [mm]	4.45 (2.05–8.45)	5.19 (2.33–13.2)		
TDE [mm]	4.34 (0.54–14.0)	5.56 (0.5–19.5)	7.0 (0.44–25.8)	9.70 (1.5–27.9)

The TDE for LSMD has significantly lower values than the other three methods (*p* value $< 0.05$ for ANOVA comparing LSMD with LSM, PWA, and AF)

LSMD and LSM achieved a significantly smaller mean distance than the other two methods, and a Hausdorff distance with an error within 1 mm; they therefore outperformed the other two methods in terms of matching volumetric regions. Regarding the Laplacian of the displacement field in the LSMD and LSM methods, in the right of Fig. 5, the LSDM had smaller values than LSM, which means that a smooth deformation with reduced unstable surface matching can be performed using LSMD.

#### Target displacement error

**Fig. 6:**
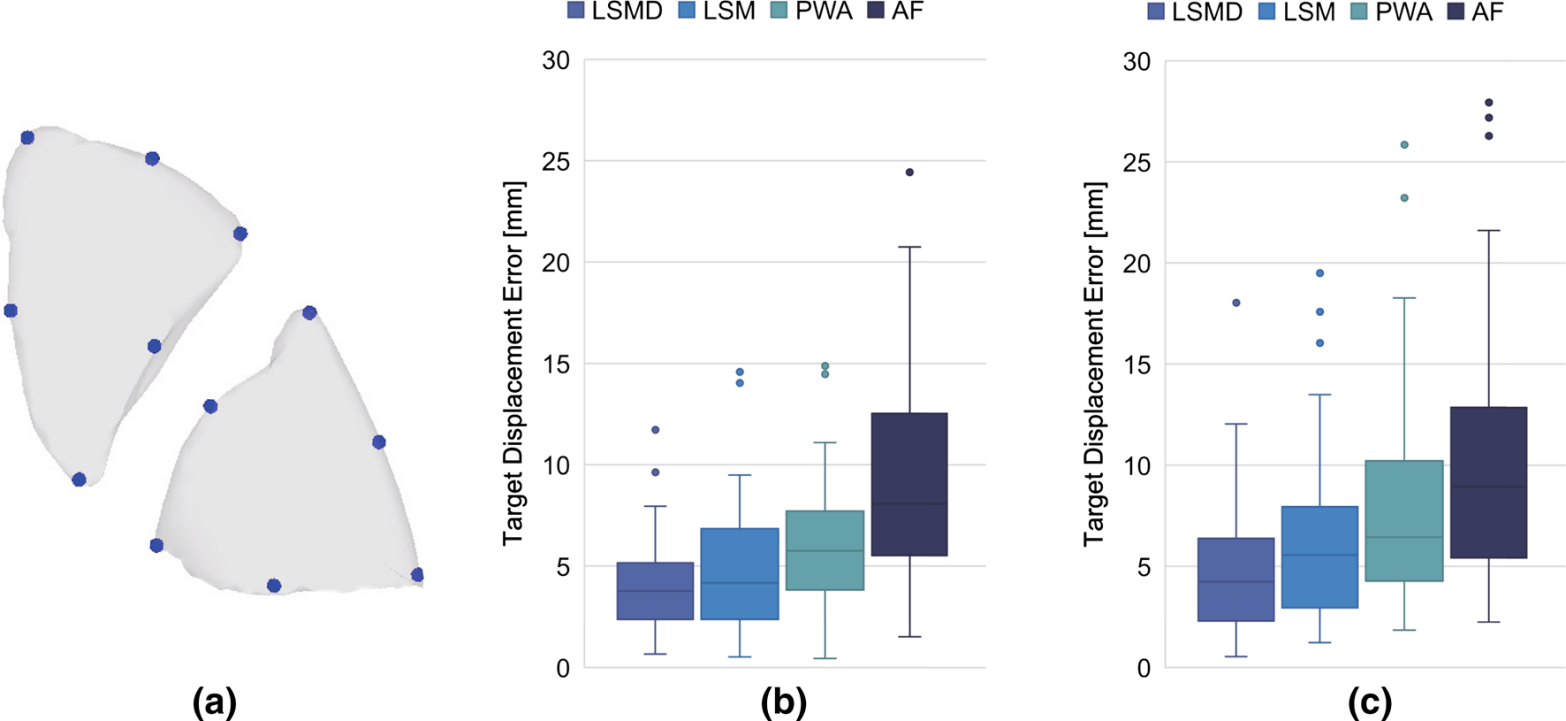
Quantitative comparison results of target displacement errors (TDE). **a** The 12 evaluation points manually indicated at the tip or ridge region in upper/lower lobes, **b**, **c** box plots of TDEs on upper and lower lobes respectively

In addition to the above geometrical indices, the accuracy of displacement vectors obtained after shape matching was evaluated by the target displacement error (TDE) defined in Eq. (7)

7 $$\begin{aligned} TDE = | \hat{\varvec{u}}_e- {\varvec{u}_e} | \end{aligned}$$

where ${\varvec{u}_{e}}$ is the displacement vector computed from the pair of the corresponding vertex of the registered inflated lung surface and deflated surface, and $\hat{\varvec{u}}_e$ is the ground truth displacement obtained from corresponding evaluation points. In this study, the evaluation points were manually placed at the three tip regions and the three midpoints on ridges in the upper/lower lobes, as shown in Fig. 6a, where relatively large deformation and incorrect matching can be assumed.

Figure 6b, c shows the results of the quantitative performance analysis of the registration accuracy on the datasets of the 11 subjects. The error in the lower lobes was larger than that in the upper lobes, which is consistent with the anatomical characteristics, with the volume change and deformation of the lower lobes being generally large. The average, minimum, and maximum of the TDEs of all datasets for LSMD, LSM, PWA, and AF are summarized in Table 2. The TDE of LSMD was significantly lower than that of the other three methods (*p* value $< 0.05$ for ANOVA test comparing LSMD with LSM, PWA, and AF). The results show that the proposed method can provide localized displacement with an error within 5 mm that it can overcome the instability problem inherent in Laplacian-based shape matching of distant structures and that it performs well for large-scale deformations. We note that TDE strictly evaluates the point-to-point correspondence and generally shows larger values than Hausdorff distance. Specifically, as in this study, the evaluation points were located at the tips or ridges of the lobes, and the registration error in the other areas is expected to be smaller than that defined by these TDEs.

### Pneumothorax deformation analysis

#### Mean and variation of deformation

No study has investigated the impact of inter-subject variation on pneumothorax deformation. Our shape matching framework can directly provide a statistical representation of the registered lung models $(M_{I}^{(k)}, M_{D}^{(k)})$, which represents the mean and variation of the pneumothorax deformation between subjects.

**Fig. 7:**
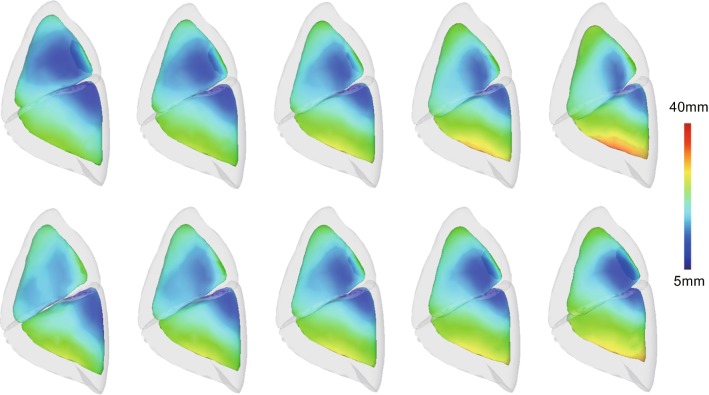
Visualizations of deformation variations corresponding to the first two eigenvalues of the obtained pneumothorax deformation models. The color map shows the magnitude of the displacements between inflated and deflated lungs. Mean deformation is located in the middle

**Fig. 8:**
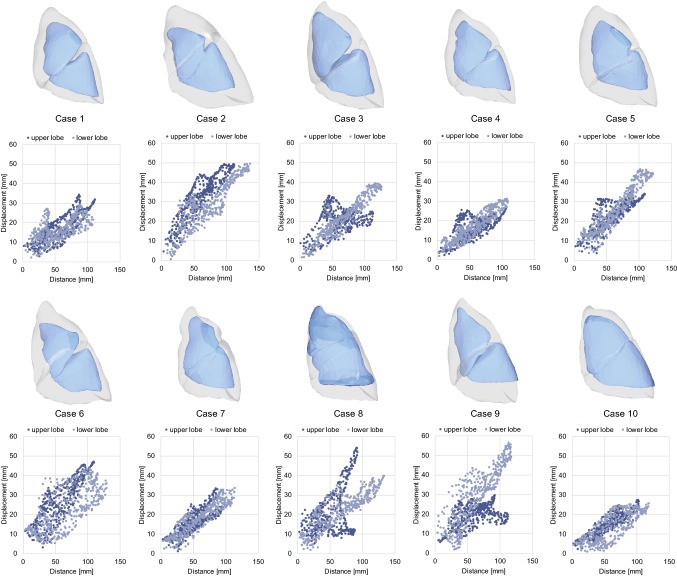
Shape matching results and the relationship between surface displacement and Euclidean distance from the hilum. The translucent image shows the inflated state and the opaque image the deflated state. In the scatter plot, the light blue and dark blue plots are the displacements of the upper lobe and lower lobe, respectively

Figure 7 shows the deformation modes that correspond to the first two eigenvalues of the obtained pneumothorax deformation models $D^{(k)}$. The eigenvalues and eigenvectors were computed from the set of displacement vectors of all vertices based on singular value decomposition. Five images were generated by changing the weights to plus/minus two times the squared root of the eigenvalues. Please see the supplemental movies that visualize the sequential motion expressed by interpolation of the inflated and deflated states. The color map shows the spatially distributed magnitude of the displacements ${\varvec{u}_i}$ between two surfaces. For example, the surfaces in the mean deformation model deform from 0–32 mm. The types of variation in the pneumothorax deformation can be confirmed according to their morphological properties as follows:

- The first eigenvector mainly encompasses variation in the scale of the deformation.
- The second eigenvector is associated with the directions and rotations of the deformation.

We also confirmed that the subspace representation using two eigenvectors explains 96.5% of the total deformation variation.

#### Linearity of deformation

Figure 8 shows the shape matching results and the relationship between the Euclidean distance from the pulmonary hilum of the upper/lower lobes and the magnitude of the displacement at all vertices composing the ten lung models (Cases 1–10). Case 11 was removed from this analysis because of incorrect pressure control during CT imaging. The number of the plots in the graph equals the number of vertices in the registered template model. The graphs show that the relationship between the distance and displacement is mostly linear; however, the displacement in the region distant from the hilum tends to be large and nonlinear. Several cases (e.g., Cases 3, 5, 8, and 9) present more complex patterns with the plots being split into two branches; this means that the deformation contains rotational components around the pulmonary hilum (as a rotation center) or bending, as shown in the right image of Fig. 1. These findings suggest that the pneumothorax deformation modeling requires both global shape changes with the assumption of linearity, and subject-specific physical interactions or boundary conditions between the lobes and the thoracic cavity.

## Discussion

To our knowledge, this study is the first to show the impact of spatial displacement on pneumothorax deformation of in vivo whole lungs within a 5-mm registration error. Past analyses have mainly focused on the internal structures of lungs measured using CBCT, and existing registration methods tended to result in large registration errors, specifically around the tips of the lobes. By integrating optimization of a differential displacement field into Laplacian-based shape matching, the proposed framework addresses problems with matching surfaces that have large curvatures and deformations and thereby achieved smaller registration errors.

To clarify the focus of this research, the displacement of the internal structures was considered to be outside the scope of this paper. Our experiments concentrated on calculation and analysis of the spatial displacements of lung surfaces, including the tips of the upper/lower lobes with large curvatures. In vivo data analysis of the internal structures of inflated/deflated lungs was reported in [26]. Non-rigid registration of lungs deformed by the patients’ postures was also investigated in [25]. For clinical applications, intraoperative iatrogenic manipulation, which can result in considerable deformation of soft tissue, should also be accounted for. To investigate this issue, [8] and [10] studied compensation mechanisms in laparoscopic surgery. Integrating biomechanical models [14, 15] with the proposed statistical models could be a solution to improve clinical applicability. As future work, we are considering a clinical application of the statistical models for VATS to CBCT data acquisition. Despite the limited measurement area, low dose CBCT imaging [25] is clinically feasible and will be useful for understanding intraoperative pneumothorax deformations in real patients. The building of statistical pneumothorax models will be an important and interesting topic for a variety of medical image analysis and intraoperative guidance research.

One technical limitation of this study is that the registration scheme relies on surface matching and does not evaluate the effectiveness of dense pixel information. As mentioned in “Introduction” section, CT intensity and image contrast changes between the inflated and deflated states are caused by differences in the air content of the lung parenchyma, which is a drawback of image-based registration. However, recent studies have reported that regularized keypoint matching improves deformable registration in lung CT and shows good scores for COPD registration [13]. Learning-based methods using convolutional networks [40] might be useful for registering subject-specific large deformations. We note that the computation time for registering each lobe was $67.6\pm 16.5$ s. The calculation cost of searching for the positional constraints is high, which restricts the number of vertices in the template. It would be interesting to integrate newer image-based registration concepts with shape matching and to evaluate their performance with the measured pneumothorax data, with the goals of facilitating precise statistical modeling and improving computation time.

Regarding the limitations of the validation protocol, surface-to-surface measures such as the Hausdorff and mean distances may be insufficient to evaluate local correspondence, especially in cases where the posture of the lobes varies greatly in the temple, or in low-curvature areas where the vertices are sparsely placed. In addition, target displacement error was evaluated on manually selected points in the high curvature areas. We consider that a further exploration of better validation protocols, such as a phantom study, is needed for more reliable evaluation.

In our experiments, the imaging data were only collected from 11 subjects, and further acquisitions were difficult because of renovations to our animal experiment facilities. Because intraoperative CT imaging is not a standard clinical protocol in thoracoscopic surgery, it is not easy to construct a patient-specific image database of collapsed lungs. However, despite this study’s limited data size, the results show that variations in pneumothorax deformation are not large. This suggests that even if there are variations in the shapes and volumes of individual lungs in the inflated state, subject-specific deformation can be formulated with relatively customized nonlinear models. This paper targeted the left lung, to measure stable pneumothorax deformation from the limited number of live dogs available. In the right lung, the physical interaction between the three (upper, middle, and lower) lobes and the ventilator may be more complex than that in the left lung. As the same measurement protocol and registration algorithms can be applied to right lungs, further research to develop statistical models of right lungs is desirable.

## Conclusion

This study aimed to analyze three-dimensional surface displacement in pneumothorax deformation using model-based shape matching techniques. To perform shape matching for substantially deformed lung images, a complete Laplacian-based shape matching solution that optimizes the differential displacement field was introduced. Our experiments showed that the proposed concept addresses problems with matching surfaces that have large curvatures and deformations, and that it achieved smaller registration errors than other techniques, even at the tip region, with spatial displacement of the lung’s surface being visualized within a 5-mm error. The findings and inter-subject statistical representations obtained in this study are directly available for further research on pneumothorax deformation modeling. In future work, we will explore deformation estimation methods and develop an intraoperative guidance system for VATS.

## Electronic supplementary material

Below is the link to the electronic supplementary material.

Supplementary material 1 (mpg 5964 KB)

Supplementary material 2 (mpg 6226 KB)
